# Thrombocytopenia and Associated Factors in Neonates Admitted to NICU during Years 2010_2011

**Published:** 2013-01-22

**Authors:** Z Eslami, M.H Lookzadeh, M Noorishadkam, A Hashemi, R Ghilian, A PirDehghan

**Affiliations:** 1 Department of Pediatrics,ShahidSadoughi University of Medical Sciences and Health Services, Yazd, Iran.; 2 2.Department of Pediatrics, Hematology, Oncology and Genetic Research Center, Shahid Sadoughi University of Medical Sciences and Health Services, Yazd, Iran.; 3 Department of Internal Medicine, Shahid Sadoughi University of Medical Sciences and Health Services, Yazd, Iran.; 4 Shahid Sadoughi University of Medical Sciences and Health Services, Yazd, Iran.

**Keywords:** Thrombocytopenia, Intensive Care Units, Neonatal, Incidence

## Abstract

**Background:**

Thrombocytopenia is the most common hematological abnormality which is encountered in the neonatal intensive care unit (NICU). The incidence in neonates varies greatly, depending upon the population studies. According to the frequency of thrombocytopenia and its complications and because of lack of such research in Iran, this study was performed on neonates admitted to Shahid Sadughi NICU during years 2011-2012.

**Materials and Methods:**

In a retrospective study, 350 neonates who were admitted to NICU were enrolled in the study. They were categorized to three groups regarding platelet count: mild, moderate and severe thrombocytopenia. Incidence of thrombocytopenia was determined and contribution of variables such as sex, gestational age, intrauterine growth retardation, asphyxia, sepsis, necrotizing enterocolitis, blood group, placental insufficiency in Gestational Diabetes Mellitus (GDM) and hypertension (HTN) were analyzed.

**Results:**

Neonatal thrombocytopenia was found in 100(28.5%) of 350 subjects, consisted of 75.3% early onset and 24.7% late onset, which most of them (96.5%) had mild and moderate thrombocytopenia, and just 3.5% had developed severe thrombocytopenia. Thrombocytopenia was associated with sepsis, intrauterine growth retardation sepsis, asphyxia, GDM, maternal hypertension and prematurity. There was no relation between occurrence of thrombocytopenia and gender.

**Conclusion:**

The incidence of neonatal thrombocytopenia was 28.5 %. Significant maternal risk factors that lead to thrombocytopenia were HTN and preeclampsia, while risk factors of neonates were asphyxia, sepsis and Intera Uterus Growth Retardation.

## Introduction

Neonatal thrombocytopenia (platelets <150 ×10^9^/litter) is one of the most common hematological abnormalities in neonates occurring in 1 to2% of healthy term neonates. The preterm or sick neonates tend to develop thrombocytopenia. Among these neonates, the incidence of thrombocytopenia is 18 to 35% (1, 2).

Of neonates admitted to neonatal intensive care units (NICUs), the platelet count drops below 150 ×10^9^/L in one in four babies and to below 50 × 10^9^/L in one in twenty (3). A large population studies showed that more than 98% of term neonates born to mothers with normal platelet counts have platelets above 150 × 10^9^/L at birth (4, 5). There is a newer classification of Neonatal thrombocytopenia (NT) based on the timing of onset of thrombocytopenia (early, within 72 h of birth versus late, after 72 h of life). These are more useful for neonatal clinicians and will help to facilitate systematic studies to improve the management of NT. Early onset thrombocytopenia is commonly associated with pregnancy complications such as intrauterine growth restriction, HELLP syndrome (hemolysis, elevated liver enzymes, and low platelet count), maternal diabetes or drug use. Clinically, the most common cause of severe early NT is known as neonatal alloimmune thrombocytopenia purpura (NAITP). However, NAITP accounts for only a small proportion (<5%) of early NT overall. NT occurs in a large proportion of preterm infants, although the thrombocytopenia is self-limiting. It usually disappeares within 10 days (6,7). The most common causes of late NT are sepsis and necrotizing enterocolitis (>80% of cases) (8,9). This form of NT which usually develops very rapidly over 1 to 2 days, is often very severe (platelets <30 × 109/L) and takes 1 to 2 weeks to recover. Such babies frequently require repeated platelet transfusion (8). In most cases, neonatal thrombocytopenia is mild to moderate and can be resolved without intervention. Life-threatening bleeding or intracranial hemorrhage (ICH) with a high risk of neurodevelopmental impairment may occur in severe thrombocytopenia (platelets <50 ×10^9^/L). Alloimmune thrombocytopenia is associated with a comparatively high bleeding risk. Late onset thrombocytopenia is typically more severe than early onset disease and bleeding is more common (10). Thus, appropriate diagnostic and therapeutic management is necessary to prevent death or neurological sequelae in the severely thrombocytopenic neonate.

The prevalence of neonatal thrombocytopenia is unknown in Iranian populations. Hence, current study was undertaken to evaluate the prevalence and causes of thrombocytopenia in neonates.

## Materials and Methods

A retrospective study was conducted on 350 neonates who admitted to NICU Shahid Sadoughi Hospital in Yazd, Iran. Cord blood samples were obtained for platelet (PLT) counts and preparation of blood smear slides. Informed consent forms were obtained from mothers of neonates with thrombocytopenia. These forms included maternal and neonatal information. Maternal information were about diabetes, hypertension, eclampsia and Preeclampsia, autoimmune disease, drug history during pregnancy and RH blood groups. Neonatal information were about gestation age, IUGR (intera uterus growth retardation), asphyxia, sepsis, RH blood groups, chief complain and final diagnosis during admission.

PLT counting was performed on ethylene diamin etetra acetate- anticoagulated blood with a standard automatic blood cell counter. Thrombocytopenia was defined as PLT counts of lower than 150 × 10^9^ per L, whereas moderate and severe thrombocytopenia was defined as less than 100 × 109 and fewer than 50 × 109 per L .Early thrombocytopenia was defined from birth to 72hr and late after 73hr of birth. 

**Statistical Analysis**

The data was summarized and analyzed using SPSS 14.0 statistical software. Student’s t-test and chi-square test were used to analyze. The results were expressed as means and standard deviations. A P-value of less than 0.05 was taken as significant.

## Results

We recorded that, during the 1-year period, 350 neonates were admitted at the NICU of Shahid Sadoughi Hospital. One hundred (28.5%) of cases were thrombocytopenia. Fifteen of thrombocytopenic samples were not analyzed due to incomplete maternal and neonatal information. Therefore, a total of 85 cord blood samples were suitable for the study, representing 51(60%) of neonates were female and 34(40%) male. Forty two cases (49.4%) of patient had mild and 40 cases (47.1%) moderate and 3 (3.5%) of the neonates were sever thrombocytopenia. Sixty four (75.3%) patients were with early onset and 21(24.7%) cases were with late onset of thrombocytopenia. Thrombocytopenia was present in 56(65.9%) of preterm and 29(34.1%) of term neonates.

Thirty eight (59.4%) of with early onset thrombocytopenia were mild and 26(40.6%) were moderate. None of them was severe thrombocytopenia. In the late thrombocytopenia group, 42(49.4%) were mild and 14(66.7%) moderate, and 3(14.3%) were sever thrombocytopenia. (P-value=0.112) 

The most common maternal factor was maternal hypertension (46.4%) (Table I) and the most common neonatal factor was sepsis (Table II). 

In the early oncet patients, 28.8% of neonates had sepsis, 23.1% IUGR, 17.3% asphyxia, 3.8% IUGR and sepsis, 9.6% IUGR and asphyxia, 1.9% sepsis and asphyxia, and 1.9% had ABO incompatibility. None of them had NEC, and 13.5% had other etiology. In the late onset patients, 40% had sepsis, 15% IUGR, 5% asphyxia, 10% ABO incompatibility, 10% NEC, and 20% had others etiology (P-value= 0.95). In the early onset patients, 52.2% of mothers had hypertension, 30.4% diabetes, 13% hypertension and diabetes, and 4.3% had eclampsia. In the late onset patients, 20% had hypertension, 40%diabetes, 20% eclampsia, and 20% had ITP (P-value=0.103).

In Patients with mild thrombocytopenia, 29.4% had IUGR, 23.5% sepsis, 20.6% asphyxia, 11.8% IUGR and asphyxia, 2.9% IUGR and sepsis, 2.9% sepsis and asphyxia, and 8.8% had other etiology. In Patients with moderate thrombocytopenia, 11% had IUGR, 38.9% sepsis, 8.3% asphyxia, 2.8% IUGR and asphyxia, 2.8% IUGR and sepsis, 8.3% ABO incompatibility, 5.6% NEC, and 22.2% had other etiology. Fifty percent of patients with severe thrombocytopenia had IUGR and 50% had sepsis (P-value=0.321). In patients with mild thrombocytopenia, 46.7% of mothers had hypertension, 33.3% diabetes, 13.3% hypertension and diabetes, and 6.7% had eclampsia. In Patients with moderate thrombocytopenia, 60% of mothers had hypertension, 20% diabetes, 10% hypertension and diabetes, and 10% had eclampsia. In Patients with severe thrombocytopenia, 66.7% of mothers had diabetes and 33.3% had ITP (P-value=0.132).

**Table I:** Incidence maternal risk factor in thrombocytopenia neonate

Maternal Risk Factor	Frequency	
	N	%
HTN	13	46.4
GDM	9	32.1
GDM+HTN	3	3.6
Eclampsia	2	7.1
ITP	1	3.6

**Table II:** Incidence neonatal risk factor in thrombocytopenia neonate

Neonatal risk factor	Frequency	
	N	%
Sepsis	23	31.9
IUGR	15	20.8
Asphyxia	10	13.9
Asphyxia+IUGR	5	6.9
Sepsis+IUGR	2	2.8
Asphyxia+Sepsis	1	1.4
NEC	2	2.8
ABO	3	4.2
Other	11	15.3
Sum	72	100

## Discussion

Thrombocytopenia is a frequent challenge for neonatologists, as it affects 22 to 35% of infants admitted to the neonatal intensive care unit. Multiple diseases can cause neonatal thrombocytopenia and these can be classified as that inducing early thrombocytopenia (≤72 h of life) and those inducing late-onset thrombocytopenia (N≥72 h) (11).The incidence of thrombocytopenia in neonates varies significantly, depending on the population studied. In this review, we found 100 (28.5%) neonates in NICU that were thrombocytopenic. In a similar study which was conducted by Henry E and coworkers on 807neonate admitted in NICU of MC Master University, 22% of neonates were thrombocytopenic (12). 

On the other hand, in a study that performed by Naguri MH and coworkers on 258 neonates in NICU, 70% was thrombocytopenic. In another study, in Nigeria by Jeremiah Z and coworkers on 132 neonates that admitted in NICU, 53% were thrombocytopenic (14). But in another study in Indonesia the incidence of thrombocytopenia was lower. In this study that was conducted by Kusamsari N and coworkers 12% of neonates in NICU were thrombocytopenic (15). 

In our study, 75.3% of neonates were early onset, and 24.7% were late onset. This is similar to study of Jeremiah Z et al. In their results 84.4% were early onset and 15.6% late onset (14). In contrast with finding of Henry E, our investigation showed that 49.4% of neonates had mild thrombocytopenia, and 47.1% had moderate and , 3.5% of them had sever thrombocytopenia. In their study, 42% of neonates had mild thrombocytopenia, 38% and 20% of them had moderate and sever thrombocytopenia, respectively. Compared to their findings, the number of patients with severe thrombocytopenia was lower in our study (12). 

Similar to other studies we did not find any significant differences in the incidence of thrombocytopenia in both genders (16,17,18,19).

We found that the incidence of thrombocytopenia were twice in preterm neonates than term neonates, this is similar to results of Bonifacio et al (17). 

This study showed, the most common maternal factors were hypertension and diabetes,that caused early onset thrombocytopenia while neonatal factors were asphyxia, sepsis and IUGR .Although NEC had low frequency, it could cause severe thrombocytopenia. This is similar to finding of Robert I et al. They reported hypoxia, diabetes, hypertension and IUGR which caused early onset thrombocytopenia, and ABO incompatibility and NEC as late onset thrombocytopenia (18).

## Conclusion

Neonatal thrombocytopenia is a common clinical problem in NICU. We ensure accurate diagnosis and to determine the most maternal and neonatal factors can reduce neonatal mortality and morbidity. 
